# Supplementary material for: Environmental risk factors for self-harm during imprisonment: A pilot prospective cohort study
Source: PLoS One. 2025 Feb 4;20(2):e0318200. doi: 10.1371/journal.pone.0318200 (PMC11793820; doi:10.1371/journal.pone.0318200)
Supplement: Attachment — Submitted filename: Response to reviewers.docx [file pone.0318200.s003.docx]

[Thomas.stephenson@kcl.ac.uk](mailto:Thomas.stephenson@kcl.ac.uk)

1^st^ June 2024

Dear Dr Ismail,

Thank you for the opportunity to revise our manuscript in relation to reviewer’s comments. Alongside our submission of a revised manuscript, in this letter we have outlined our response to each point raised by the reviewers. Our responses are in italics.

# Points raised by reviewer #1

## Abstract and introduction

### 1

The introduction lacks contextualizing of self-harm during a life-course in antisocial, aggressive and/or offending populations. Self-harm is more likely to have occurred both before and after imprisonment. Furthermore, self-harm is overrepresented in acute phases (e.g., remand), acute psychiatric conditions (e.g., adjustment disorders), and generally enduring psychiatric disorders (e.g., anxiety, personality, and severe mental disorders). Thus, the introduction lacks information regarding self-harm as part of the psychiatric spectrum in prisoners and consequently lacks preceding information about psychiatric morbidity in prison populations. I believe this omission reflects a conscious choice from the authors. However, I would argue that this information is crucial in their introduction, and it seems the authors also consider it important, as evidenced, for example, by the inclusion of such details in their questionnaires.

Response: *We are grateful to the Reviewer for this observation and have amended the introduction to provide further acknowledgement of the importance of these factors which, we agree, are crucially important to understanding self-harm behaviour within these populations. Thus, we have included the following within the second paragraph of the Introduction: “These include self-harm behaviours prior to imprisonment and pre-existing psychiatric morbidity (for example, personality and depressive disorders). High levels of such morbidity have been documented across prison populations in many countries.” We have not, in this case, cited a link between remand status and self-harm behaviours as this association was not found in the recent meta-analysis we have cited.*

### 2

The second sentence of the introduction, which mentions the troublesome doubling of self-harm incidents among prisoners. This substantial increase prompts questions about the report’s methodology and the reasons behind this trend. Furthermore, the citation of a non-academic article as the source raises concerns about the claim’s credibility (I have not reviewed the given reference). It would be beneficial to substantiate this claim with research from academic sources and from various contexts.

Response: *We thank the Reviewer for this observation. In our view, the data referenced are credible because the UK Ministry of Justice have standardised data collection and reporting mechanisms across prisons in England and Wales that record self-harm incidence. There is little epidemiological data of comparable size available from non-governmental sources. We have additionally cited the last available findings on prevalence of self-harm amongst men and women in a national prevalence study across England and Wales based on data from 2004-2009 in the first sentence of the Introduction section, in support of the claim that self-harm is a major public health issue in prisons.*

### 3

The final sentence of the first paragraph, which describes self-harm as a resource burden for service providers, lacks citations. Moreover, its relevance to the paper may be questioned. If the authors deem this information important to the introduction, further elaboration and supporting references are needed, but may also be saved for the discussion and clinical implications of the study.

Response: *The resource burden associated with self-harm is of relevance to the clinical services responsible for the mental health care of prisoner populations. The care planning process used to monitor those at increased risk of self-harming behaviour, the Assessment, Care in Custody and Teamwork process (ACCT), has significant staff resource implications for both prison and clinical services. We agree that this point is better placed in the Discussion: Clinical implications sub-section: it is now moved and reworded as the second sentence in this section of the paper as follows: ‘Any reduction in self-harming behaviour occasioned by the suggested environmental changes (e.g., reduced use of single cell placements) has the potential to improve prisoner well-being and to reduce the use of resource intensive care planning processes such as the Assessment, Care in Custody and Teamwork (ACCT) process employed in prisons in England and Wales.’*

### 4

The last sentence of the second paragraph requires improvements in language and clarity, along with more precise reporting of the odds ratio, including a confidence interval.

Response: *We thank the Reviewer for noting this.* *No confidence interval was reported for this variable. We have reflected on the difficulty the reader might have when interpreting this data, despite the addition of other data reported in the study such as the Χ^2^ value and p value. We have opted to remove quoting of any OR value. Therefore, we have rephrased this to read “One case-control study additionally identified that frequent cell moves within prison over the previous two years was associated with self-harm behaviour^5^.”*

### 5

In the third paragraph, the authors discuss a wide array of prison environmental factors that might contribute to self-harm, but the direction of these associations should be clarified. Furthermore, while the authors note the current uncertainty around the strength of these effects, it is important to recognize that the strength of associations varies significantly depending on the context and prison population. This aspect warrants some elaboration.

Response: *We have clarified the direction of each of the relationships implicated in the wider literature. Whilst we fully acknowledge the Reviewer’s point that the strength of associations is likely to vary depending on context and prison population, we have reserved our acknowledgement of this point for the discussion as we think it is of greater relevance when discussing the value of further replication studies than in introducing this single-site study. Thus, it is addressed in the fourth sentence of the Discussion: Future research sub-section as follows: ‘Replication of the study findings in research in prisons of varying security, location and function, and in the female prison population, will improve generalisability and allow for cross-comparison of the strength of effects.’*

### 6

The introduction incorporates multiple references to specifically female samples, which is not inherently problematic. However, given that this study focuses on an all-male sample, it is important to clearly highlight when employing such references.

Response: *We thank the Reviewer for this observation and have acknowledged this by amending the third paragraph of the introduction as follows: “In addition, most existing studies examine female prisoner populations. Longitudinal study designs could provide high-quality evidence regarding these relationships in both male and female populations, which could in turn inform the clinical assessment and management of self-harm in this population and suicide prevention strategies in prisons.”*

### 7

The aims appear to be structured as a primary and a secondary aim, suggesting a hierarchy of importance. If my understanding is correct, this distinction should be explicitly stated.

Response: *As suggested by the Reviewer, we have removed to secondary aim. Please see response to Intro point 8 below.*

### 8

The introduction provides minimal context regarding the secondary aim. This aim might be considered for removal. Alternatively, it would be beneficial to elaborate on the design of a wider array of previous studies (including sample size, and statistical approaches), and the insights they have offered for the purpose of this aim.

Response: *As suggested by the Reviewer, we have removed the secondary aim.*

### 9

Mentioning that the article was part of a wider project is redundant for the aims paragraph. However, as a reader, I would appreciate a summary description of the SHAPE project (if this is what the authors are referring to here) in the methods section, but I leave this entirely up to the authors to decide.

Response: *We have moved this and provided a summary description of the SHAPE project in the Procedures and measures sub-section of the Methods section.*

## Methods

### 1

Please describe the attrition, for example, how many participants were excluded due to their English language skills or decision-making capacity, respectively.

Response: *We are grateful to the Reviewer for requesting further detail here. We have amended the text from the fourth sentence of the second paragraph onwards to separate those not included (n=147) and those excluded on records-based and in-person screening and provide details of the breakdown in each group. We have made the corresponding edits to figure 1.*

### 2

Participants lost due to suicide during the follow-up period should be mentioned, even if there were no cases, if this was measured.

Response: *We have clarified that no participants died by suicide during follow-up by adding a sentence at the end of the second paragraph of the Results – Cohort characteristics sub-section.*

### 3

Category B prisons are contrasted with Categories C and D but should also be contrasted with Category A.

Response: *We have added the phrase “and a lesser emphasis on security than those in Category A” to the second sentence of the Methods: Sample sub-section.*

### 4

A strength of this study is that the sample corresponds to the general prison population on key factors. However, note observed differences between prisoners awaiting trial, those sentenced, and those detained under immigration law in the final sample.

Response: *We have amended the first paragraph of the ‘Results – Cohort characteristics’ sub-section to reflect the change in proportion of those on remand and sentenced in the final cohort.*

### 5

It is unclear whether the sample comprises “all male prisoners who had recently arrived” or an all-male sample.

Response: *We thank the Reviewer for highlighting this potential source of confusion.* *It is stated in the first paragraph of the Methods section that the study site is a men’s prison. Thus, we have rephrased the first sentence of the second paragraph to remove any further references to sex, to avoid confusion. It now reads “The study population consisted of all prisoners arriving into the study prison between 17 March and 16 July 2022.”*

### 6

Briefly describe the simple random sampling approach used in this study.

Response: *Our sampling approach involved a series of weekly random samples during 18 weeks of recruitment between March to July 2022. We have amended the text in the third sentence of the second paragraph of Methods: Sample sub-section to clarify this.*

### 7

If I understand Figure 1, the sentence describing eligibility criteria in the sample segment is need of correction. Should it state “aged 18 or older, male, and current prison spell had lasted less than one month”?

Response: *We thank the Reviewer for spotting this error in classification of the length of current prison spell as an exclusion criterion and have corrected it to classify ‘having a current prison spell lasting less than one month’ as an inclusion criterion. We have made the corresponding edits to figure 1 and adjusted the numbers included/not included and excluded/not-excluded accordingly.*

### 8

The average length of follow-up was 73.5 days. Please indicate the standard deviation or other measure of variability.

Response: *We have added the standard deviation, 24.1 days, to the manuscript.*

### 9

The instruments used such as DUDIT and CTQ, are insufficiently described and not referenced. Furthermore, it is not clear how information from these instruments were utilized in this study and if they were administered alongside the supplemented questionnaires.

Response: *We are grateful to the Reviewer for the opportunity to clarify this point.* *Although these instruments were administered alongside the study questionnaire, data collected using them were not used in the present study. Amongst the data used in this study, data for one confounder factor (violent offence history) and one exposure factor (vape use) were assessed in the baseline interview. The remaining data collected in the baseline interview were for use in a separate study within the SHAPE project which is in preparation. We have therefore amended the text to reflect this and have moved the first two paragraphs of the sub-section ‘Procedures and measures’ to be below the summary description of the SHAPE project (see response to Intro 9). We have renamed this sub-section ‘Measures and procedures’ to reflect the order in which these are now described in the revised paragraph.*

### 10

In the methods section, it is mentioned that this was an exploratory pilot study, a description with which I agree. However, the language in the abstract, aims, and discussion needs revision to consistently reflect this.

Response: *We have adjusted the descriptions of the study throughout the manuscript to consistently reflect this.*

### 11

The three participants who withdrew their consent were not accounted for in the comparison of non-identifiable demographic data, as they seem to be missing from the stated n=126, referencing the last paragraph in the sample segment. This figure should likely be adjusted to 129 and comparative test rerun accordingly.

Response: *We have adjusted this figure to n=129 and rerun the comparative tests.*

### 12

Throughout the document, ensure that statistical notation is used correctly, such as changing ‘mean’ to italicized M, and consistently reporting odds ratios with or without the 95% numeral in accordance to applied reporting standards.

Response: *We thank the Reviewer for this observation. We have amended the manuscript throughout to include statistical notation where appropriate (i.e. in Tables and when reporting numerical results with a mathematical operator). However, we have retained the use of terms rather than symbols in the narrative text to improve readability. This is consistent with APA style guidelines. Nevertheless, we are very happy to defer to the Editor for the final decision here. We have addressed the reason for not presenting the confidence intervals for the OR quoted in the introduction (now removed; see Intro point 4). We consistently report 95% CI for other odds ratios throughout the manuscript.*

### 13

The independence of the researchers from the regular prison staff should be clarified in the methods section. If there was no independence, this raises questions for the limitations section, such as the potential impact on informed consent and the data collected. Additionally, the lack of researcher blindness to participants could have influenced the findings. The potential effects of this potential lack of blinding and independence on the study’s reliability and impartiality need to be addressed.

Response: *We have clarified that researchers were independent from the regular prison staff (final paragraph of the Methods: Measures and procedures sub-section). In the penultimate paragraph of this sub-section, we have clarified that TS was blinded to all participants’ exposure values except for vape Use at the time of carrying out the exit interviews.*

### 14

SHAPE is used as an abbreviation but not explained.

Response: *This is now explained as per response to Intro point 9.*

### 15

The decision to include vape use as an exposure for self-harm warrants further explanation. Since this variable has been singled out based on the authors’ unpublished results, it is crucial to offer a rationale for its inclusion. Additionally, clarifying why vape use is considered an environmental risk factor, rather than a risk factor related to individual behaviour, is needed.

Response: *We have now clarified that this variable was included, alongside Covid-19 in-cell isolation, based on the unpublished results of the parallel qualitative study, by including it in the summary of the preliminary results of this study (see response to Methods point 21) in the first paragraph of the Methods: Measures and procedures sub-section. We have outlined the rationale for this exposure being considered as an environmental risk factor in the final sentence of the same paragraph, as follows “Vape use was included and considered as an environment-related variable given that participants in the authors’ parallel qualitative study cited frequent vape replacement issues as a contributing factor towards self-harm behaviour.”*

### 16

It is unclear whether number of cellmates includes a cumulative number of cellmates the participants have during the follow-up or the number of people the prisoner is sharing their cell with at data collection.

Response: *We have clarified that this refers to cumulative number of cellmates in the first paragraph of the Methods: Measures and procedures sub-section. For additional context, we have also added a sentence to the first paragraph under the Methods: Sample sub-section describing that the study prison contains a combination of double occupancy and single occupancy cells.*

### 17

I would like confirmation that risk factors were assessed at baseline and not during the three-month follow-up period, as assumed from the prospective design, but this is not entirely clear.

Response: *Exposures were assessed during follow-up as outlined in paragraphs 1 and 2 of the Methods: Measures and procedures sub-section except for vape use which was assessed during the baseline interview (see response to Methods point 9). We have amended the wording of paragraph 2 of this sub-section so that sentences 4 and 5 now read “Vape use was assessed by self-report at baseline interview. All other exposures were measured using routinely collected data on participants’ NOMIS prison record during follow-up.”*

### 18

The structure of variables such as the number of cell changes, number of cellmates, time to first phone call, time on the phone, emergency bell response rate, prisoner-staff ratio, and prisoner-listener ratio could be reconsidered, for example through standardization or dichotomization.

Response: *We thank the Reviewer for this suggestion. We did consider categorisation of some of these variables; however, they were all suitable for use in the analyses in their unadulterated forms and for ease of interpretation by the reader we decided to retain their original scales.*

### 19

The methods state that TS and IH conducted interviews while the roles of researchers state TS, IH, and CA, correct or clarify this please.

Response: *We have amended the Roles of Researchers section to clarify that TS and IH did all interviews whilst TS, IH and CA carried out data extraction.*

### 20

Studies in preparation are generally referred to and referenced in the reference list.

Response: *We have amended the manuscript to reference studies in preparation throughout (two in total in revised manuscript, see response to Methods point 9).*

### 21

Describe the methods of the scoping review and briefly describe the preliminary results from the parallel qualitative study you apply in this study.

Response: *The* s*coping review involved examining the findings of two recent literature reviews, including a systematic review & meta-analysis of individual-level risk factors for self-harm (Favril et al., 2020) and a literature review of the relationship between prison regime characteristics and adverse outcomes including self-harm (Stephenson et al., 2021). We have clarified this in the second sentence of the first paragraph in the Methods: Measures and procedures sub-section. We have added the preliminary findings from the qualitative study which fed into predictor selection, which included that “low staffing numbers, staff non-responsivity to distress, restrictive regime characteristics, vape replacement issues and COVID-19 prevention measures contribute to self-harm.”*

### 22

Ethnicity is used both as a categorical variable in describing attrition and as a dichotomized variable in regression analysis. Please choose one approach for consistency.

Response: *We have dichotomised ethnicity throughout and have re-run the attrition-related chi squared tests comparing ethnicity of the cohort with decliners and the general prison population.*

### 23

The exit interview timing is unclear, as it is stated to be conducted after the end of the follow-up period, which is hard to conceptualize as to me the exit interview would represent the end of follow-up. This process was said to occur approximately 4 weeks after completion, in parenthesis it mentions 7 days. Please clarify the exact timing of the exit interviews and what actually represents the end of follow-up for consistency and understanding.

Response: *Data collection in this follow-up study was chiefly records-based, with an adjunct exit interview included to assess the primary and secondary study outcomes and one exposure. We have clarified this in the sixth paragraph of the Methods: Measures and Procedures sub-section of the manuscript as follows: “The end of follow-up was defined as the end of participants’ third month in prison and no data was extracted from their records after this point. At the exit interview, participants were asked about outcomes and exposures occurring during the follow-up period only (i.e., any self-reported outcome occurring after the end of three months was discounted).”*

### 24

The authors state that they have included their secondary outcome, NSSI ideation, with a view to improving the statistical power. I disagree with this. Instead, they have studied two quite different outcomes (i.e. NSSI and NSSI ideation). In regards to power, the power would be increased by, for example, adding more participants to the study, the statistical tests employed, or changing the significance level – not by analysing a distinct outcome.

Response: *On reflection, we agree with the reviewer that these are related but distinct outcomes and we have thus removed the reference to improving statistical power.*

### 25

A (recent) history of self-harm and/or suicide attempts is captured in the baseline questionnaire and may represent a confounder for several analyses in this study, as mentioned in the limitations in regards to single cell and prisoner-staff ratio. This information could be used in sensitivity analyses.

Response: *We agree with the Reviewer’s comment that past history of self-harm and/or suicide attempt is a potential confounder. When those with prior self-harm/suicide attempt are removed from the analyses, the total outcome counts for both study outcomes (1/55 for self-harm behaviour and 6/53 for ideation) are too low to produce meaningful results. Thus, we have opted not to use this factor in sensitivity analyses but acknowledge it as a limitation in the Discussion section.*

### 26

In the light of the aims it is not clear what the 66 participants that dropped out during follow-up adds to the study. This should be clarified or reframe the study as a study of a sample of 83/82 individuals.

Response: *We thank the Reviewer for highlighting this.* *We have clarified in the results section of the abstract the proportion and number of participants who completed follow-up.*

### 27

It is stated that the authors did not analyse solitary confinement as a predictor. However, such results are presented in Table 5.

Response: *We are grateful to the Reviewer for noticing this error which we have corrected by removing the results for the secondary outcome presented in Table 5. It was not our intention to present these results, given the number in the exposed group was so low (2/82).*

### 28

The high levels of missingness in time on phone-variable require methods to deal with this issue or exclude it from analysis.

Response: *We have excluded this variable from the analysis due to high levels of missingness. We have removed the corresponding results from the revised Table 4 (see response to Figures and tables point 2).*

### 29

The authors state that they conduct multivariable logistic regression models. However, it seems to me as if the unadjusted models are univariable. This requires clarification.

Response: *We have amended the first sentence of the third paragraph of the Methods: Statistical analysis sub-section to clarify this.*

### 30

It is confusing that the authors state that they use a set p-value cut-off, yet they consider a positive tendency (CI 0.93 – 7.75, p=0.071) to be associated with the outcome.

Response: *We are grateful to the Reviewer for noticing this error and have removed it from the fourth paragraph of the Methods: Statistical analysis sub-section.*

## Figures and tables

### 1

Table 2 generally displays that those lost to follow-up differ in few aspects, aspects already mentioned in the results section. Mentioning this in text would suffice. Thus, removing some information from the table. This will improve its readability without losing any relevant information.

Response: *We have removed this column and the column of those who declined to participate from Table 2.*

### 2

Tables 3 and 4 should be combined into a single table, with clear indications of the units for each variable (e.g., minutes, days, and percent). The table should, as currently presented, denote missing data. However, the accompanying text can then draw attention to the key findings and significant aspects of the data presented in this consolidated table rather than levels of missingness.

Response: *We have combined these tables as suggested and included text presenting significant features of the data in the text of the third paragraph of the Results: Cohort characteristics sub-section.*

### 3

For readability, S3 Supplementary table 1 is better summarized in text and not as a supplement.

Response: *We have removed the supplementary table as requested as this information is already summarised in the text in the second paragraph of the Results: Cohort characteristics sub-section as follows: “The event rate for any self-harm episode from records during follow-up was 17.1% (14/82). This was comparable to the rate assessed from self-report (13/78, 16.7%). […] The corresponding event rate for any self-reported self-harm ideation was 28.2% (22/78).”*

## Results, discussion, and conclusions

### 1

Key information in regards to the validity of the secondary aim is the correlation or concordance or NSSI and NSSI ideation and could be reported. Auxiliary, the comparison between the 14/82 registered versus the 13/78 self-reported events of NSSI suggests a need to assess their concordance or discordance as suggested by the authors in the methods section. This also have bearing on the third limitation.

Response: *We have added text to the second paragraph of the ‘Results – Cohort characteristics’ sub-section on the discordance between the records-based and self-reported self-harm behaviour events (6.1% of cases), and on the concordance between records-based self-harm behaviour and self-reported self-harm ideation. We are grateful to the Reviewer for highlighting this and the implications for further research. We have amended the final sentence of the Discussion: Future research sub-section to read “Discordance between outcomes assessed via prison medical records and self-report was low (6.1%) but may nonetheless have implications for future study designs in this area.”*

### 2

The baseline questionnaire lacks questions about the incidence of self-harm/suicide attempts during the ongoing legal process, focusing instead solely on occurrences within prison at any given time. Including this information is essential for a prospective study, an issue that is briefly mentioned in the limitations section.

Response: *We acknowledge the importance of assessing self-harm behaviour in the lead-up to imprisonment and include this as a consideration for future work in the fourth sentence of the Discussion: Future research sub-section as follows: “Future research should seek to address in-study aggression and recent self-harm behaviour (for example, self-harm occurring in the period between initial arrest and imprisonment) as a potential confounders of relationships between prison environment exposures and self-harming outcomes.”*

### 3

I am concerned about the methodology used by the authors in treating the staff-to-prisoner ratio variable, specifically the imputation of numbers based on prison conditions in a new ward. This may have influenced the negative association found for prisoners per staff member. Without direct access to the data, it is impossible to evaluate this issue. Therefore, I recommend the authors to carefully consider this factor and potentially reanalyse their data. This concern is heightened by the observation that a previously significant relationship turned insignificant after the exclusion of just one outlier, casting further doubt on the validity of the results regarding the staff-to-prisoner ratio.

Response: *We thank the Reviewer for outlining their concern regarding imputation of missing data for this variable.* *Staffing data was missing across all areas of the prison for 1 day during 218 days of follow-up. The day in question was a Monday, and we dealt with the missing data by imputing the M of the staffing values for the two most comparable days i.e., the previous and following working-day Mondays. We had to skip back two weeks in this case, because the Monday one week before the day in question was a Bank Holiday with distinctly lower staffing numbers. In our view, the imputation of <0.5% of the data is not likely to explain this result, and alternative methods for dealing with this missingness, such as excluding participants whose exposure values included this imputed data, are not necessary. We provide what we think is a more plausible explanation for the finding i.e., reverse causality. Furthermore, while the sensitivity analysis in question resulted in a non-significant association with staff-prisoner ratio, the direction of the relationship was unchanged.*

### 4

Remove the term "unexpected" in relation to the negative association found between staff-prisoner ratio and NSSI, as the article present no hypotheses and is framed as explorative.

Response: *We have removed this term.*

### 5

Given the low number of participants and the relatively few positive primary outcomes, it is advisable to handle outliers through methods other than simple removal. Techniques such as Winsorization could be considered to explore or manage extreme values and its effects on the robustness of the results.

Response: *We thank the Reviewer for this suggestion. As the Reviewer is aware, the data from these types of populations frequently contain extreme values that are all, potentially, clinically relevant. We chose the strategy of including extreme values in the analyses (and removing them in follow-up sensitivity analyses) to retain a more complete representation of the variability of the data, rather than adjusting these values based on an arbitrary threshold to make our standard errors appear smaller.*

### 6

My interpretation is that the results does not indicate a major issue of power regarding interpretations of time in activities, working status and social visit status. An issue of power would have been indicative by a tendency in a given direction but with a too wide confidence interval to draw conclusive results. However, conceptually the issue of power is of course relevant here, as in any small sample study, but not based on the presented findings.

Response: *Thank you to the Reviewer for the observation*. *We have amended the fourth paragraph of the Discussion section to reflect that the findings in question do not indicate a major issue of statistical power.*

### 7

In the limitations section, you mention that the study size was too small to detect small to moderate effect sizes. Yet, you have identified some small to moderate effect sizes in the current study. This needs clarification.

Response: *We agree and have amended the first paragraph of the Discussion: Limitations sub-section to discuss the impact of a low event count on the precision of some of the estimates.*

### 8

Length of stay up to baseline could have had influence on the risk factors that was not accounted for and is a limitation. Furthermore, it is a limitation that information regarding the length of follow-up was not incorporated in statistical analyses and could be mentioned.

Response: *A theoretical limitation is that the baseline interview often did not take place until a few weeks after entry, however the only data presented in this study that was collected at baseline interview is the confounder of past offending history, measured by self-report (see response to Methods point 9). As this is a historical variable, in our view the length of stay up to baseline would not have had any influence on it. We back-dated the beginning of records-based exposure assessment to the date of entry to the prison, after participants were recruited, to compare the same period for all participants. We then followed them all up to a set time-point, regardless of the date of baseline assessment. The length of follow-up for all participants who completed follow-up was therefore the same (3 months i.e., 13 weeks i.e., 91 days).*

### 9

Given the authors discussion of aggression and single cell placements, your discussion, for example, in regards to the second limitation, would benefit from considering the context of existing studies on aggression (separate from self-directed aggression) as a factor linked to self-harm.

Response: *We have amended this section to refer to findings in a recent meta-analysis identifying assault perpetration as a potential a risk factor for self-harm.*

# Issues raised by reviewer #2

None.

# Summary

Thank you for the opportunity to revise this manuscript. We have resubmitted the manuscript with tracked changes (entitled ‘Revised Manuscript Tracked Changes’) and without (entitled ‘Revised Manuscript’). We have also resubmitted two versions of all figures and tables that have been edited (with and without track changes; Figure 1 and Tables 2-4). Please note, of those tables not resubmitted, supplementary table 3 is to be removed from the submission following the reviewers’ comments.

Kind regards,

Dr Tom Stephenson

On behalf of and with agreement of all authors
