# Supplementary material for: Environmental risk factors for self-harm during imprisonment: A pilot prospective cohort study
Source: PLoS One. 2025 Feb 4;20(2):e0318200. doi: 10.1371/journal.pone.0318200 (PMC11793820; doi:10.1371/journal.pone.0318200)
Supplement: Attachment — Submitted filename: Response_to_reviewers_auresp_2.docx [file pone.0318200.s004.docx]

[Thomas.stephenson@kcl.ac.uk](mailto:Thomas.stephenson@kcl.ac.uk)

2 November 2024

Dear Dr Ismail,

Thank you for the opportunity to revise our manuscript further in relation to the comments in the first reviewer’s second review. Alongside our submission of a manuscript with further revisions, in this letter we have outlined our response to each point raised in the reviewer’s second review. We have used the reviewer’s numbering of comments (1.3, 2.1 etc) but added a letter indicating the section of the paper (e.g. A1.3 for Abstract and introduction) to avoid confusion. Our responses are in italics.

**General comments**

**Reviewer’s comment:** I would like to express my gratitude to the editor for the opportunity to review this revised article and to the authors for the significant improvements they have made. The authors have effectively addressed most of my previous comments and have provided well-reasoned explanations for the areas where they chose not to make revisions. However, I still hold one major concern (see R1.17) regarding the analytic approach and a few instances where my queries remain unsatisfactorily addressed. I have detailed these below (for reference, "R1.3" pertains to the third specific comment in my previous review, while "R2.1" refers to new specific comments in this review).

Generally, I would like to invite the authors to revise the study to better reflect the APA guidelines (or the chosen guideline to be followed consistently). For example and based on my understanding, “(17/218)” should be written as “17 out of 218” (or simply as a percentage). Additionally, the number of decimal places varies across the manuscript, and the numerical representation of thousands should be consistent (i.e., 1,000 or 1000). Tables should be capitalized in text and numbers that never exceed one should not be written with a zero (i.e., p = .03 is correct, while p = 0.03 is incorrect according to APA). I also noticed a missing “p=” in the first paragraph of the regression analyses. Furthermore, the text could benefit from minor edits to improve clarity and reduce ambiguity. The tables, in particular, require such editing. For example, the sentence regarding the exclusion of participants in the sample section needs revision, and there is a repetition of “due to [with subsequent information]” in the same section.

**Response:** *We are grateful to the reviewer for highlighting discrepancies in the manuscript, and have now corrected these throughout. Regarding the presentation of numbers, we have applied the PLOS statistical reporting guidelines throughout the manuscript. Our responses to the numbered points raised are included in the sections below.*

**Abstract and introduction**

Comment A1.3

**Reviewer’s comment in first review:** The final sentence of the first paragraph, which describes self-harm as a resource burden for service providers, lacks citations. Moreover, its relevance to the paper may be questioned. If the authors deem this information important to the introduction, further elaboration and supporting references are needed, but may also be saved for the discussion and clinical implications of the study.

**Response:** *The resource burden associated with self-harm is of relevance to the clinical services responsible for the mental health care of prisoner populations. The care planning process used to monitor those at increased risk of self-harming behaviour, the Assessment, Care in Custody and Teamwork process (ACCT), has significant staff resource implications for both prison and clinical services. We agree that this point is better placed in the Discussion: Clinical implications sub-section: it is now moved and reworded as the second sentence in this section of the paper as follows: ‘Any reduction in self-harming behaviour occasioned by the suggested environmental changes (e.g., reduced use of single cell placements) has the potential to improve prisoner well-being and to reduce the use of resource intensive care planning processes such as the Assessment, Care in Custody and Teamwork (ACCT) process employed in prisons in England and Wales.’*

**Reviewer’s comment in second review:** If available, use an academic or other reference for this intervention.

**Response:** *We have not identified any academic reference for the ACCT process, however we have added references to two publicly available documents on the UK Ministry of Justice website which outline this process.*

Ministry of Justice*. Prison Service Instruction 64/2011: Management of Prisoners at Risk of Harm to Self, to Others and from Others (Safer Custody). London: Ministry of Justice; 2011.* [Available online at <https://www.gov.uk/government/publications/managing-prisoner-safety-in-custody-psi-642011>, accessed on 21/10/24.]

Ministry of Justice. *Annex to PSI 64/2011 (ACCT). London: Ministry of Justice; 2020.* [Available online at <https://www.gov.uk/government/publications/managing-prisoner-safety-in-custody-psi-642011>, accessed on 21/10/24].

*See response to comment R2.3 regarding changes the content of the text where these references are cited.*

Comment A2.1

**Reviewer’s comment in second review:** The fourth sentence in the second paragraph of the introduction, starting with “[t]he authors…”, seems to use reference (4) but this is not clear.

**Response:** *We are grateful to the reviewer for spotting this ambiguity and have updated the wording of that sentence to read “The same systematic review and meta-analysis also identified…”.*

**Methods**

Comment M1.17

**Reviewer’s comment in first review:** I would like confirmation that risk factors were assessed at baseline and not during the three-month follow-up period, as assumed from the prospective design, but this is not entirely clear.

**Response:** *Exposures were assessed during follow-up as outlined in paragraphs 1 and 2 of the Methods: Measures and procedures sub-section except for vape use which was assessed during the baseline interview (see response to Methods point 9). We have amended the wording of paragraph 2 of this sub-section so that sentences 4 and 5 now read “Vape use was assessed by self-report at baseline interview. All other exposures were measured using routinely collected data on participants’ NOMIS prison record during follow-up.”*

**Reviewer’s comment in second review:** To my understanding, the study collected data on certain risk factors at baseline, but with most risk factor information gathered during the prospective follow-up period. Further, the data for the outcome of self-harming behaviour was also collected during this follow-up period. Logistic regression analyses were conducted, without considering follow-up time or time-to-event in the statistical analyses.

In a prospective cohort study, it is possible to analyse risk factors collected during the same follow-up period as the outcome. However, this approach has notable limitations, such as the risk of reverse causality, as the authors are aware. Understanding cause and effect – or as in here, when estimating effect sizes – requires proper time orientation, i.e., an exposure (risk factor) must occur before the outcome. This also requires having a sufficient level of detail in the data regarding when exposure and outcome occur so that the temporal order can be accurately distinguished (or explored/estimated). Furthermore, it requires an adequate model of analysis. The authors briefly discuss reverse causality as a limitation, but I find this to be insufficient. I recommend that the authors improve their analytic approach pertaining to the regression analyses approach. This could involve using alternative methods or multiple methods that consider the varying characteristics of risk factors, and reanalyse their data. Favourably, with time-to-event and, if appropriate, subsequent censoring taken into account. If my understanding is correct, approximate time-sensitive data is available to the authors through prison medical records. This would significantly improve the article. If the authors decide to revise their analytic approach, I further recommend that the authors reconsider the number of risk factors included in the analysis of the outcome. Given the low number of participants and events, the current number of risk factors may be too extensive, even for an exploratory pilot study.
These revisions are important partly due to the authors’ allusive recommendations on prison services based on interpretations of the data that are currently weakly supported by the data due to mentioned limitations. Furthermore, the authors state and acknowledge that a self-harm event increases the probability of encountering certain risk factors after the event, making the timing of data collection even more crucial when employing risk factors in statistical analyses collected before and after potential events.

**Response:** *Due to limited time resources, we did not plan to collect time-sensitive data for exposure factors in this pilot and so planned logistic regression analyses between exposure factors and binary outcomes instead. Unfortunately, alternative methods involving collection of time-sensitive data are now no longer available to us, due to loss of access (via NOMIS custodial records) to the exposure data for participants after they move on from the study prison. We are grateful to the reviewer for emphasising the importance of the limitation this analysis method places on any conclusions about causality in the relationship between exposure factors and outcomes.*

*We have thus added the following sentence to the second paragraph of the Methods: Measures and procedures sub-section to signpost the lack of time-to-event data and justify the choice of methods, and hope the reviewer agrees that this provides sufficient signposting that the subsequent acknowledgement of it as a limitation in the Discussion section is sufficient:*

*“Due to limited resources, the timing of exposures and outcomes was not assessed in this study.”*

*We have also edited the fourth sentence of the Discussion: Future research sub-section to read “Future studies assessing time-to-event data could help to discern the direction of any effects underlying the associations* *observed” with the intention of further emphasising the above.*

*To emphasise the limited scope of this pilot study, we have amended the manuscript title to include the term “pilot study” and have added to the study’s aims an estimation of the attrition rate in the study population to inform future research.*

*We have removed the recommendations for prison services in the clinical implications sub-section (see response to comment R2.3) in further acknowledgement that this is pilot data which requires replication.*

*For the reviewer’s information, we are now funded by NIHR to conduct a larger prospective cohort study (with sample size calculations based on strength of associations observed in this pilot) where we plan to analyse time-to-event data for a smaller set of exposure variables using survival analysis approaches. We are grateful to the reviewer for his/her helpful thoughts in this area which helped to strengthen our application and analytic approach.*

Comment M2.1

**Reviewer’s comment:** In the first paragraph of the sample section, the authors state that previous longitudinal research at the same study site has documented a higher than average prevalence of self-harm, suggesting it is a suitable study location. However, this suitability depends on the context. While it may offer valuable insights, it could also potentially lower the generalizability of the findings, depending on the study’s aims. This increased prevalence might be influenced by systematic biases, such as the characteristics of the sample or the prison environment. Please consider this and revise accordingly.

Additionally, as a reader, I am interested in the previous research mentioned. If the authors deem it appropriate, I would recommend including a reference to this prior research.

**Response:** *We agree with the reviewer that the suitability of a study location depends on several contextual factors and have clarified in that paragraph that we refer to suitability “in terms of feasibility for a pilot study”.*

*We have acknowledged the potential impact of systematic biases referred to in the reviewer’s comment by adding the following to the third paragraph of the Discussion: Limitations sub-section:*

*“The study was conducted in a prison with a higher-than-average prevalence of self-harm, and this higher prevalence may be influenced by unmeasured systematic biases such as the characteristics of the prison environment. Such biases would lower the generalisability of findings to other prison settings and sub-populations.”*

*The prior research informing our decision about study location is now referenced in the manuscript (reference 14).*

**Figures and tables**

Comment F2.1

**Reviewer’s comment:** The tables, especially Table 2 and 3 needs improvement, focusing on removing redundant information, minimizing double reporting in text and tables, as well as general readability.

**Response:** *We thank the reviewer for highlighting issues with the manuscript tables. We have made several changes across tables 2, 3 and 4 to address the points raised:*

- *Removed redundant text in column labels*
- *Harmonised terminology across tables and manuscript e.g. self-harm episode corrected to self-harm behaviour*
- *Removed dual unit labels, separating parameters into new rows where necessary (e.g. SD and IQR in table 3)*
- *Removed redundant information, e.g. Missing data rows where no data missing for that variable*
- *Rearranged variables for readability, e.g. Prisoner-staff ratio and Emergency bell response rate moved below Prisoner-Listener ratio in table 3 because we report M and SD for these variables rather than Mdn and IQR as for variables above*
- *Formatted of numbers brought into line with changes made across the manuscript to present max 2dps, e.g. p values in table 4 reformatted.*

**Results, discussion, and conclusions**

Comment R1.1

**Reviewer’s comment in first review:** Key information in regards to the validity of the secondary aim is the correlation or concordance or NSSI and NSSI ideation and could be reported. Auxiliary, the comparison between the 14/82 registered versus the 13/78 self-reported events of NSSI suggests a need to assess their concordance or discordance as suggested by the authors in the methods section. This also have bearing on the third limitation.

**Response:** *We have added text to the second paragraph of the ‘Results – Cohort characteristics’ sub-section on the discordance between the records-based and self-reported self-harm behaviour events (6.1% of cases), and on the concordance between records-based self-harm behaviour and self-reported self-harm ideation. We are grateful to the Reviewer for highlighting this and the implications for further research. We have amended the final sentence of the Discussion: Future research sub-section to read “Discordance between outcomes assessed via prison medical records and self-report was low (6.1%) but may nonetheless have implications for future study designs in this area.”*

**Reviewer’s comment in second review:** The information of discordance is important. However, the paragraph with this information could do with streamlining.

**Response:** *We thank the author for highlighting that this paragraph was in need of streamlining. We have now edited it down for clarity so that it now reads:*

*“The event rate for any self-harm behaviour during follow-up was 17.1% (14/82) based on medical records and 16.7% based on self-report (13/78), with discordance between the two measures in 5 of 78 (6.1%) cases for whom data was available such that 19.5% of participants (16/82) had a positive classification for self-harm behaviour on at least one measure. The event rate for any self-reported self-harm ideation was 28.2% (22/78). The concordance between self-harm behaviour outcome from records and self-harm ideation outcome from self-report was 82.1% (64/78) amongst those for whom data was available for both measures. No participants died from suicide during follow-up.”*

Comment R1.3

**Reviewer’s comment in first review:** I am concerned about the methodology used by the authors in treating the staff-to-prisoner ratio variable, specifically the imputation of numbers based on prison conditions in a new ward. This may have influenced the negative association found for prisoners per staff member. Without direct access to the data, it is impossible to evaluate this issue. Therefore, I recommend the authors to carefully consider this factor and potentially reanalyse their data. This concern is heightened by the observation that a previously significant relationship turned insignificant after the exclusion of just one outlier, casting further doubt on the validity of the results regarding the staff-to-prisoner ratio.

**Response:** *We thank the Reviewer for outlining their concern regarding imputation of missing data for this variable.* *Staffing data was missing across all areas of the prison for 1 day during 218 days of follow-up. The day in question was a Monday, and we dealt with the missing data by imputing the M of the staffing values for the two most comparable days i.e., the previous and following working-day Mondays. We had to skip back two weeks in this case, because the Monday one week before the day in question was a Bank Holiday with distinctly lower staffing numbers. In our view, the imputation of <0.5% of the data is not likely to explain this result, and alternative methods for dealing with this missingness, such as excluding participants whose exposure values included this imputed data, are not necessary. We provide what we think is a more plausible explanation for the finding i.e., reverse causality. Furthermore, while the sensitivity analysis in question resulted in a non-significant association with staff-prisoner ratio, the direction of the relationship was unchanged.*

**Reviewer’s comment in second review:** Thank you for a good response. However, here I believe that the authors have misunderstood parts of my comment, in part based on my comment’s ambiguity. My stated concern was primarily regarding the methodology used regarding the prison wing that was closed at the start of the study (i.e.: One prison wing was closed at the start of the study but gradually reopened during the second half of the study and reached approximately 50% capacity at end of the follow-up period. The total occupancy for this particular wing was imputed as 25% of capacity throughout the period of reopening based on consultation with prison officials). Please reconsider this comment and my apologies if I am misunderstanding the given response.

**Response:** *We thank the reviewer for bringing the potential impact of this assumption to our attention and apologise for misunderstanding the original comment. We have now conducted a sensitivity analysis for this imputation. Data for the numerator for this ratio variable (i.e. the total number of prisoners placed on the wing) were imputed for a total of 5/82 participants who spent time on the wing, equivalent to 17/1066 participant-weeks within the dataset (given 13 weeks follow-up per participant). The mean prisoner-staff ratio for all participants (31.88, n=82) changed little when excluding participants placed on this wing (32.09, n=77). The mean ratio for those excluded was lower, at 28.63 (n=5).*

*Compared with our findings for the whole cohort, where an increase in the prisoner-staff ratio of 1 was associated with an OR of 0.89 for any self-harm behaviour (p=0.04, n=82), a sensitivity analysis which excluded those placed in the partial-capacity wing did not change the association observed between the two variables, and if anything resulted in a stronger association, with an OR of 0.87 for any self-harm behaviour (p=0.02, n=77). We have included the result of this sensitivity analysis in the manuscript in the Results: Sensitivity analyses sub-section.*

Comment R2.1

**Reviewer’s comment:** As the aim was to “…estimate the effect sizes…”, the discussion lacks discussion regarding the effect sizes in the current study. Please revise the discussion to include this, or alternatively, adjust the aim if this better align with the study’s underlying purpose.

**Response:** *Given that many of the exposures tested in the study are novel, we hope the reviewer agrees that the consideration, as it is, of the effect size estimates in comparison with findings in the existing literature (for example, regarding single cell placement) is sufficient.*

Comment R2.2

**Reviewer’s comment:** The discussion appropriately highlights the novelty of several results, which is commendable. However, it appears to lack adequate contextualization of these findings within the specific setting of the study, such as the remand context, the prison environment with a higher-than-average prevalence of self-harm, the male sample, and the location in England. Providing a more detailed contextualization of the results could enhance their relevance and usefulness, after considering comment R1.17.

**Response:** *We have amended the first sentence of the Discussion section to include these characteristics in the summary of the study with the intention that this context is then clear in the subsequent paragraph outlining the novel aspects of the study design.*

*We have additionally amended the sentence on replication in the Discussion: Future research sub-section so that it includes mention of replication in settings with varying prevalence of self-harm, so that it now reads:*

*“Replication of the study findings in research in prisons of varying security, location, function and with varying prevalence of self-harm, as well as in the female prison population, will improve generalisability and allow for cross-comparison of the strength of effects.”*

Comment R2.3

**Reviewer’s comment:** In the clinical implications section, it is (kind of) mentioned that the authors suggest a reduced use of single-cell placements. This either needs to be explicitly stated as a recommendation from the authors or revised. My interpretation of the results, in combination with the discussion above this section, is that the results are preliminary and in need of replication before making such recommendations, partly as the findings may vary across different subgroups and contexts. Therefore, such a recommendation may currently be most valuable to the specific prison in question and for guiding future studies at this time. Furthermore, this revision should also reflect the R1.17-comment. Nevertheless, I leave the final interpretation and wording up to the authors.

**Response:** *We have removed the recommendation regarding the reduction in single cell placement and reworked this sentence so that it now reads:*

*“If replicated, the relationships observed in this study between self-harm and several prison environment factors may have implications for practice in the assessment and management of self-harm by clinical and non-clinical staff. This may also have system-level implications for mental health promotion measures in prisons and for resources spent on intensive care planning processes such as the Assessment, Care in Custody and Teamwork (ACCT) process employed in prisons in England and Wales^26,27^.”*

*We agree with the reviewer that the results are preliminary and have emphasised this by reworking the first sentence of the Discussion: Future research sub-section so that it now reads:*

*“The limitations above make the results of this study preliminary and in need of replication in better powered longitudinal studies.”*

Other changes

We have replaced all mentions of the term “self-harm episode” with “self-harm behaviour” for consistency.

We have amended the study title and acronym to include reference to it being a pilot project.

We have added the values for *n* for the sensitivity analyses in the Results: Sensitivity analyses sub-section.

We have streamlined the sentences on novelty of the study within the Discussion section for readability.

We have removed the words “problems with” from the final sentence of the Discussion: Limitations sub-section.

Summary

Thank you for the opportunity to revise this manuscript further. We have resubmitted the manuscript with tracked changes (entitled ‘Revised Manuscript Tracked Changes’) and without (entitled ‘Revised Manuscript’). We have also resubmitted two versions of the tables that have been edited (tables 2-4) with and without tracked changes.

Kind regards,

Dr Tom Stephenson

On behalf of and with agreement of all authors
